# The Potential Applications of Peroxisome Proliferator-Activated Receptor *δ* Ligands in Assisted Reproductive Technology

**DOI:** 10.1155/2008/794814

**Published:** 2008-12-16

**Authors:** Jaou-Chen Huang

**Affiliations:** Division of Reproductive Endocrinology and Infertility, Department of Obstetrics, Gynecology & Reproductive Sciences, University of Texas Health Science Center at Houston, Houston, TX 77030, USA

## Abstract

Peroxisome proliferator-activated receptor *δ* (PPAR*δ*, also known as PPAR*β*) has ubiquitous distribution and extensive biological functions. The reproductive function of PPAR*δ* was first revealed in the uterus at the implantation site. Since then, PPAR*δ* and its ligand have been discovered in all reproductive tissues, including the gametes and the preimplantation embryos. PPAR*δ* in preimplantation embryos is normally activated by oviduct-derived PPAR*δ* ligand. PPAR*δ* activation is associated with an increase in embryonic cell proliferation and a decrease in programmed cell death (apoptosis). On the other hand, the role of PPAR*δ* and its ligand in gamete formation and function is less well understood. This review will summarize the reproductive functions of PPAR*δ* and project its potential applications in assisted reproductive technology.

## 1. INTRODUCTION

Assisted reproduction uses a spectrum of technologies to enhance fertility. In vitro fertilization (IVF) is the most advanced and most sophisticated assisted reproductive technology (ART). Since the first “IVF baby”, Louise Brown, was born in 1978, IVF has gradually been accepted by the general public. Nowadays, IVF is a routine procedure to treat the infertile couples. Recent data shows more than 45 000 IVF babies were born in the US each year (http://www.cdc.gov/ART/ART2005/section1.htm) and there are more than three millions “IVF babies” in the world (http://news.bbc.co.uk/1/hi/health/5101684.stm).

Compared with embryos in natural pregnancies, IVF embryos have low implantation potential (about 15–20% per embryo, http://apps.nccd.cdc.gov/ART2005/nation05.asp). In order to increase IVF success, it is common to transfer two or more embryos to the uterus. This practice not only increases the odds of pregnancy but also increases the chance of multiple pregnancy. Compared with natural conception, IVF pregnancies are thirty times more likely to be multiple (32% versus 1%, http://www.cdc.gov/ART/ART2005/sect2_fig5-15.htm#Figure 8). Furthermore, 13% of the IVF multiple pregnancies are “high-order” multiple pregnancies, that is, triplets or more. These pregnancies are prone to develop obstetrical complications and pose great risks to mothers and infants. As a result, some infants suffer short-term complications and life-long sequels. Therefore, one of the most urgent tasks in ART is to enhance the implantation potential of IVF embryos so that transferring single embryo yields an acceptable chance of success.

It has long been observed that, compared with in vivo embryos, embryos cultured in simple media develop at a slower pace [1, 2] and have more apoptosis [3]. It is generally accepted that, compared with in vivo embryos, IVF embryos are in a less optimal environment—they are not in the supportive and protective environment of the oviduct. As a result, IVF embryos do not develop to their full potential and do not implant as well as their in vivo counterparts. It has been proposed that modifying embryo culture conditions, making them similar to those of the oviduct, may improve embryo development and enhance IVF success. Recent reports show embryos express PPAR*δ* and that PPAR*δ* activation by oviduct-derived ligand enhances embryo development and implantation (more below). Thus PPAR*δ* is a novel pathway that could be exploited to enhance ART outcome. This article will review the current literature regarding PPAR*δ* and reproduction and outline the potential applications of its ligands in ART. Because PPAR*δ* interacts with PPAR*α* and -*γ*, relevant information regarding PPAR*α* and -*γ* will also be provided.

## 2. PPARS AND THEIR LIGANDS

Peroxisomes are organelles in eukaryotes that participate in fatty acid oxidation. In 1990, the first PPAR (PPAR*α*) was discovered in the mouse [4]. Two years later, PPAR*α* and two additional PPAR isotypes, PPAR*β* (also known as PPAR*δ*) and PPAR*γ*, were discovered in the *Xenopus* [5]. Subsequently, all three isotypes were found in mouse and many species including human. PPAR*δ* was originally discovered in a human osteosarcoma cell line [6] and later found to be the human homolog of PPAR*β* in the *Xenopus*.

PPARs are ligand-activated transcription factors. They form heterodimers with another nuclear receptor, retinoid X receptor (RXR), which also has three isotypes: RXR*α*, *β*, and *γ* [7]. The functions of PPAR-RXR complexes are determined by PPAR isotypes. Although either PPAR or RXR ligand can activate PPAR-RXR complexes, simultaneous PPAR and RXR binding yields more potent activities [8]. Whereas PPAR*α* and PPAR*γ* activate genes related to glucose and lipid metabolism, only a few genes are reported to be directly regulated by PPAR*δ*. PPAR*δ* reportedly upregulates PDK1, ILK [9] and 14-3-3*ε* [10], and downregulates PTEN [9] and 11 beta hydroxysteroid dehydrogenase type 2 gene [11]. The functions of these genes, unlike those regulated by PPAR*α* and PPAR*γ*, are not limited to energy homeostasis. They include carbohydrate homeostasis (PDK1), cell migration, proliferation and adhesions (ILK), signal transduction (ILK) and its modulation (14-3-3*ε*) and, finally, tumor suppression (PTEN).

Unlike PPAR*α* and PPAR*γ*, the outcome of PPAR*δ* activation is not limited to the transcriptional activities of genes directly regulated by PPAR*δ* because PPAR*δ* also modulates the transcriptional activities of PPAR*α*, PPAR*γ*, other nuclear receptors (such as estrogen receptor), and BCL-6 (a transcriptional repressor). A recent report shows that binding of PPAR*δ* by its ligand allows full transcriptional activities of PPAR*α* and PPAR*γ*, which is normally inhibited by nonliganded PPAR*δ* [12]. In addition, binding of PPAR*δ* by its ligand releases a transcription repressor BCL-6 [13] which targets a group of genes with diverse activities including transcription regulation (*n* = 18), protein binding (*n* = 11), signal transduction (*n* = 10), catalysis (*n* = 8), structural molecule activity (*n* = 3), enzyme activity regulation (*n* = 3), protein transportation (*n* = 2), cell movement (*n* = 2), chaperone (*n* = 1), and unknown function (*n* = 3) [14]. Thus PPAR*δ* interacts with an extensive array of intracellular proteins to regulate cellular functions.

A diverse group of chemicals including hypolipidemic drugs, herbicides, and industrial plasticizers causes liver tumors in the rodents. They induce peroxisome proliferation and led to the discovery of PPAR*α* [4]. Fatty acids, particularly the unsaturated fatty acids, and certain eicosanoids bind to PPAR*α*, -*γ*, and -*δ* with varying affinities [8, 15]. Although all PPAR isotypes bind to unsaturated fatty acids, PPAR*α* has the highest affinity. Eicosanoids from the lipoxygenase pathway (such as leukotrienes and hydroxyeicosatetraenoic acids—HETEs) and the cyclooxygenase pathway (such as prostaglandins—PGs) bind to PPARs: leukotriene B4 and 8(S)-HETE are PPAR*α* ligand, 15-deoxy-Δ12,14-PGJ_2_ (a PGD_2_ derivative) is a PPAR*γ* ligand, and PGI_2_ is a PPAR*δ* ligand [15]. In addition to the natural ligands, PPARs also respond to synthetic ligands. Some of the synthetic PPAR ligands are currently used to treat metabolic diseases: fibrates, which bind to PPAR*α*, are hypolipidemic agents; thiazolidinediones (TZDs), which bind to PPAR*γ*, are insulin sensitizers. A recent report shows that retinoic acid, depending on the ratio of cellular retinoic acid binding protein 2 (CRABP-II) and fatty acid binding protein 5 (FABP5), may function as a PPAR*δ* ligand [16].

## 3. BIOLOGICAL FUNCTIONS OF PPAR*δ*

The roles of PPAR*α* and PPAR*γ* in energy homeostasis are relatively easy to understand because the former is predominantly expressed in the brown adipose tissue and liver, and the latter, the adipose tissue [8, 17]. While PPAR*α* catabolizes lipid in the liver, PPAR*γ* facilitates fatty acid storage in adipose tissue by inducing the maturation of preadipocyte to fat cells.

The functions of PPAR*δ*, on the other hand, are not as easy to ascertain because PPAR*δ* has a ubiquitous distribution (including high levels of expression in the gut, kidney, and heart, and a lower level of expression in the liver) and interacts with extensive arrays of proteins in the cells (more in Section 2). Reported functions of PPAR*δ* include the formation of intestinal adenoma [18] and colon cancer [19], the healing of skin [20], the development of hair follicles [21], and the protection of cells against noxious stimuli [10, 22]. The reproductive function of PPAR*δ* was revealed for the first time during the investigation of cyclooxygenase-2 knockout mouse [23].

## 4. PPAR*δ* AND REPRODUCTION

In primates, including humans, mature eggs are picked up by the fimbria and become fertilized in the ampulla. The zygotes remain in the oviduct for 72 hours; develop to morula/early blastocyst stage embryos before entering into the uterus. During this period, the oviduct produces soluble factors to promote embryo development and protect the embryo.

As mentioned earlier, the link between PPAR*δ* and reproduction was first revealed at the implantation site of cyclooxygenase-2 knock out mice [23]. Since then, it was learned that embryos express PPAR*δ* and that oviducts and embryos produce PGI_2_. Recent studies also show that exogenous PPAR*δ* ligand promotes the development of embryos and enhances their implantation potential (more in Section 4.4).

### 4.1. Female reproductive tract and embryos produce PPAR*δ* ligand

We analyzed the metabolites of arachidonic acid by human [24] and mouse [25] oviducts and found substantial amount of PGI_2_. Further analysis shows that PGI_2_ production by the oviducts varies according to the estrus cycle. It peaks shortly after ovulation, coincides with the presence of cleaving zygotes in the oviduct and the “window” of embryonic responsiveness to PGI_2_ [25]. Oviducts posses PGI_2_ synthase and cyclooxygenase-2; the latter is the rate limiting enzyme of PG synthesis. The increased PGI_2_ production is due to upregulation of the cyclooxygenase-2 gene. Oviduct also produces retinoic acid [26], the effects of oviduct-derived retinoic acid on embryo development is controversial (details below).

Similar to oviducts, embryos also metabolize arachidonic acid via cyclooxygenase and lipoxygenase pathways. PGI_2_ is the most abundant metabolites of arachidonic acid by mouse embryos [27]. Preimplantation embryos express PGI_2_ synthase, and cyclooxygenase-1 and -2; all are expressed in early stage and throughout the preimplantation period. The preimplantation embryos also produce retinoic acid [28] but its role in embryo development is yet to be determined.

The uterus is known to produce PGI_2_ [29] but its central role in assisting embryo implantation was not revealed until twenty years later [23]. The uterus produces retinoic acid [30] but its biological role is unclear. Similarly, the ovary produces retinoic acid [31] and PGI_2_ [32] but the extent to which they interact with PPAR*δ* to influence oocyte maturation is not clear.

### 4.2. Testes express PPAR*δ*

All three PPAR isotypes are present in Sertoli and Leydig cells of the testes: PPAR*α* and -*δ* transcript and protein are expressed in Leydig cells and Sertoli cells of rat [33], PPAR*γ*1 transcript is detected in human testis [34], both PPAR*α* and -*γ* transcripts and proteins are expressed in mouse Sertoli cells [35]. In addition, mouse spermatids and spermatocytes express PPAR*δ* [36]. The functionality of PPAR*δ* in the testes is supported by the presence of *Ssm*, a novel PPAR*δ* target gene in mouse testes [37]. Thus PPAR*δ* may directly or indirectly (i.e., via PPAR*α* or -*γ*) affect spermatogenesis. Information regarding PPAR*δ* expression and action in mature sperms is limited. We previously report that iloprost (a PGI_2_ analog) does not affect sperm activity [38]. However, the response of mature sperms to synthetic PPAR*δ* ligand or retinoic acid has not been reported.

### 4.3. Ovaries express PPAR*δ*

Similar to testes, the ovary expresses all three PPAR isotypes [39]. While PPAR*δ* is expressed throughout the ovary, PPAR*α* is mainly expressed in the theca and the stroma, and PPAR*γ*, in the granulosa cells (of human, pig, rodents, and sheep) and the oocytes (of cattle and zebrafish). Of the three PPAR isotypes in the ovary, only PPAR*γ* shows cyclic changes thus implying its role in follicular genesis and/or oocyte maturation. Since PPAR*δ* may regulate the transcriptional activity of PPAR*γ*, the growth of follicles or oocytes may be indirectly modulated by PPAR*δ* ligand. The expression of PPAR*δ* and the effect of its activation on the oocytes remain unclear at the moment.

### 4.4. Preimplantation embryos express PPAR*δ*

Compared with PPAR*γ* [28, 40] or PPAR*α* [28, 40], there is more information regarding the expression of PPAR*δ* and the outcome of its activation on preimplantation embryos [28, 40]. Mouse embryos express PPAR*δ* at an early stage [40, 41] and throughout the preimplantation period. Blastocyst stage embryos express PPAR*δ* in the inner cell mass and the trophectoderm [40]. PPAR*δ* activation is associated with embryonic cell proliferation and improved embryo development [40]. Supplementing L-165,041 (a synthetic PPAR*δ* ligand) or iloprost (a PGI_2_ analog) to culture media enhances embryo hatching [38, 40–42]. Pretreatment with iloprost also increases the potentials of implantation and live birth of mouse embryos [40, 43].

The reported effects of retinoic acid on embryo development are inconclusive: some show it is beneficial to embryo development [44, 45], others show it is detrimental [46, 47]. A recent report shows LG100268 (a synthetic RXR ligand) reduces trophectoderm cell proliferation in a concentration-dependent manner, but enhances the development of bovine blastocysts at 0.1 *μ*M [48]. Since retinoic acid binds to retinoic acid receptor (RAR), RXR, and (depending on the balance of intracellular CRABP-II and FABP5) PPAR*δ*, the effect of retinoic acid on embryo development is likely to depend on its concentration as well as receptor availability. The latter is likely to change according to the developmental stage of the embryo. More studies are needed to resolve this complex issue.

## 5. CLINICAL APPLICATIONS OF PPAR*δ* LIGAND IN ART

PPAR*δ* can be activated via one of the three methods: PGI_2_ (either stable analog or natural PGI_2_), synthetic PPAR*δ* ligand, or retinoic acid. The data presented above supports the notion that embryonic PPAR*δ* activation by iloprost or synthetic PPAR*δ* ligand may enhance ART outcome. However, using PPAR*δ* ligand in ART should be approached with caution. A PPAR*δ* ligand suitable for ART use should have passed extensive reproductive toxicology studies involving laboratory animals as well as domestic species to assure the health of progeny. The potential applications of PPAR*δ* ligand in ART are listed below.

### 5.1. Using PPAR*δ* ligand to enhance gamete function

The potential of PPAR*δ* ligand in enhancing male gamete function or production is unknown because there is limited information on the effects of PPAR*δ* ligand on the spermatogenesis and sperm activities. PPAR*δ* may have no effect on sperm function because PGI_2_ analog does not seem to affect the motility of human sperms [38]. However, synthetic PPAR*δ* ligand or retinoic acid was not tested in the previous report.

It is not clear the extent to which PPAR*δ* ligand affects the oocytes either directly or indirectly (through granulosa cells). At the molecular level, PPAR*δ* ligand has the potential to influence follicular genesis and/or oocyte maturation via PPAR*δ* or PPAR*γ*. This influence depends on the species-specific expression of PPAR*γ* and PPAR*δ* in the oocytes and the granulosa cells [39]. Recent reports show PPAR*γ* ligand enhances the meiotic resumption of mouse oocytes [49] and reverses the adverse effects of diet-induced obesity on the oocytes [50]. More research is needed to understand the potential targets of PPAR*δ* ligand in the ovary, that is, oocyte versus granulosa cells and PPAR*δ* versus PPAR*γ*.

### 5.2. Using PPAR*δ* ligand to enhance embryo development

Three independent laboratories using different strains of mice show iloprost (a PGI_2_ analog) enhances embryo development [10, 38, 41]. A recent report, based on 60 cryopreserved human embryos donated by patients for research, confirmed the enhancing effects of iloprost on human embryos [51]. Mouse embryos previously exposed to iloprost produce more implantation sites and yield more live pups [43]. These positive findings support the use of PPAR*δ* ligand to enhance IVF outcome.

Whereas no synthetic PPAR*δ* ligand has been approved by the FDA for clinical use, iloprost (which activates PGI_2_ receptor and PPAR*δ*) is approved by the FDA to treat pulmonary hypertension and peripheral vascular disease. Iloprost has undergone rigorous toxicology test and shows no teratogenicity [52]. Various animals (including rats, rabbit, and monkeys) exposed to iloprost during the peri-implantation period and throughout the pregnancy produce normal offspring. Based on four positive results from independent sources (three involving mouse embryos and one involving human embryo described above) and reassuring reproductive toxicology profile, a small scale, phase I/II clinical trial using iloprost may be considered. Supplementing iloprost to IVF embryos mimics the environment of the oviduct which provides a PGI_2_ rich environment surrounding the embryos [24, 25].

There is a long way before retinoic acid is ready for use in ART. Given that PPAR*δ* activation by retinoic acid is dependent on the ratio of CRABP-II and FABP5, the extent to which retinoic acid functions as a PPAR*δ* ligand in the embryos is likely to vary based on the developmental stage of the embryos. More research is needed to ascertain the stage-dependent response of embryos to retinoic acid.

### 5.3. Using PPAR*δ* ligand to augment PPAR*δ* system at the implantation site

The uterus, being the site of implantation, is as important as the embryos in ensuring a good ART outcome. Therefore, although uterus is not an area served by ART, a discussion about PPAR*δ* ligand and ART is not complete without a brief discussion of the uterus.

It is PPAR*δ* at the implantation site that establishes the first link between PPAR and reproduction [23]. Recent evidence suggests that a host of signaling pathways involved in decidualization, implantation, and placentation converge on PPAR*δ*: maternal PPAR*δ* is important for decidualization and implantation; embryonic PPAR*δ* is important for placentation [53]. Thus enhancing the PPAR*δ* system of the uterus may be a novel method to ensure ART success. Activating PPAR*δ* system at the implantation site is different from activating PPAR*δ* system in the preimplantation embryos. The former involves administering PPAR*δ* ligand to potential mothers during the peri-implantation period; the latter involves exposing IVF embryos to PPAR*δ* ligand prior to embryo transfer. The former may require days of treatment; the latter only takes 18–24 hours (i.e., during the eight cell to morular stage transition). In addition, more information regarding genes and pathways activated by PPAR*δ* ligand in the uterus and their impacts on the progeny needs to be obtained before it can be used to target the uterus.

### 5.4. Using PPAR*δ* ligand to improve oocyte cryopreservation outcome

One important aspect of ART is the cryopreservation of oocytes. Oocyte cryopreservation is considered as a solution to ovarian aging in women who wish to defer raising their family; it is also viewed as one of the methods to preserve fertility in young women who are about to receive chemotherapy or irradiation. The outcome of oocytes cryopreservation is, however, far from satisfactory [54]; its success is hampered by freezing injury [55]. Lipid in the egg membrane and inside the cytoplasm is believed to be one of the contributing factors. Modifying membrane lipid [56] or removing excess cytoplasmic lipid [57] reportedly enhances oocyte survival after cryopreservation. Since PPAR*δ* regulates lipid metabolism, it may mobilize lipid and augment the viability and the developmental competence of cryopreserved oocytes. The suitability of the above method in human ART requires more research because lipid content in the oocytes varies among species [58].

## 6. SUMMARY

Competent gametes, quality embryos, and a receptive endometrium are essential elements for a viable pregnancy. The outcome of ART may thus be enhanced by improving any or all of the above elements. While it is high time to consider using PPAR*δ* ligand such as iloprost to enhance embryo development, the application of PPAR*δ* ligand in other areas of ART requires more research. Continuing research on the reproductive functions of PPAR*δ* and the safety of its ligand will ensure a smooth translation of basic science to clinical medicine.
